# (*E*)-2-Methyl-5-(thiophen-2-ylmethyl­idene)cyclopentan-1-one

**DOI:** 10.1107/S1600536811033708

**Published:** 2011-08-27

**Authors:** Abdullah M. Asiri, Abdulrahman O. Al-Youbi, Hassan M. Faidallah, Khalid A. Alamry, Seik Weng Ng

**Affiliations:** a Chemistry Department, Faculty of Science, King Abdulaziz University, PO Box 80203 Jeddah, Saudi Arabia; b Center of Excellence for Advanced Materials Research, King Abdulaziz University, PO Box 80203 Jeddah, Saudi Arabia; c Department of Chemistry, University of Malaya, 50603 Kuala Lumpur, Malaysia

## Abstract

The exocyclic C=C double-bond in the title compound, C_11_H_12_OS, has an *E* configuration. The methyl-bearing C atom in the cyclo­pentane ring is disordered over two positions with a site-occupation factor of 0.899 (8) for the major occupied site.

## Related literature

For the synthesis of 2-(2-thienyl­idene)cyclo­pentanone, see: Austin *et al.* (2007 ▶); Tsukerman *et al.* (1964 ▶).
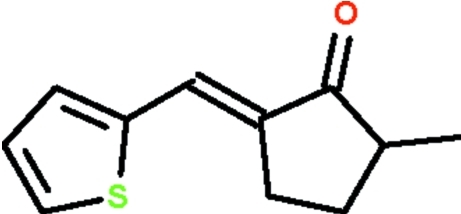


## Experimental

### 

#### Crystal data

C_11_H_12_OS
*M* *_r_* = 192.27
Monoclinic, 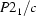

*a* = 12.0667 (5) Å
*b* = 11.0576 (4) Å
*c* = 7.3003 (3) Å
β = 100.469 (4)°
*V* = 957.85 (7) Å^3^
*Z* = 4
Mo *K*α radiation
μ = 0.29 mm^−1^
*T* = 100 K
0.25 × 0.15 × 0.10 mm

#### Data collection

Agilent SuperNova Dual diffractometer with Atlas detector
Absorption correction: multi-scan (*CrysAlis PRO*; Agilent, 2010 ▶) *T*_min_ = 0.931, *T*_max_ = 0.971
4842 measured reflections
2131 independent reflections
1817 reflections with *I* > 2σ(*I*)
*R*_int_ = 0.028

#### Refinement

*R*[*F* ^2^ > 2σ(*F* ^2^)] = 0.041
*wR*(*F* ^2^) = 0.104
*S* = 0.99
2131 reflections
122 parameters
9 restraints
H-atom parameters constrained
Δρ_max_ = 0.57 e Å^−3^
Δρ_min_ = −0.31 e Å^−3^

### 

Data collection: *CrysAlis PRO* (Agilent, 2010 ▶); cell refinement: *CrysAlis PRO*; data reduction: *CrysAlis PRO*; program(s) used to solve structure: *SHELXS97* (Sheldrick, 2008 ▶); program(s) used to refine structure: *SHELXL97* (Sheldrick, 2008 ▶); molecular graphics: *X-SEED* (Barbour, 2001 ▶); software used to prepare material for publication: *publCIF* (Westrip, 2010 ▶).

## Supplementary Material

Crystal structure: contains datablock(s) global, I. DOI: 10.1107/S1600536811033708/bt5619sup1.cif

Structure factors: contains datablock(s) I. DOI: 10.1107/S1600536811033708/bt5619Isup2.hkl

Supplementary material file. DOI: 10.1107/S1600536811033708/bt5619Isup3.cml

Additional supplementary materials:  crystallographic information; 3D view; checkCIF report

## Figures and Tables

**Fig. 1.**
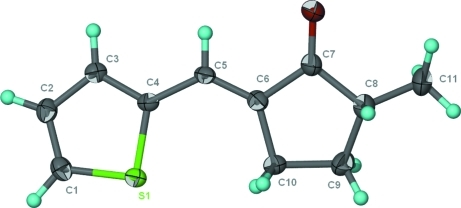
Anisotropic displacement ellipsoid plot (Barbour, 2001) of C11H12OS at the 70% probability level; H atoms are drawn as spheres of arbitrary radius. The disorder in the methine carbon is not shown.

## supplementary crystallographic information

### Comment

The α-methylene hydrogen of cyclic ketones can be abstracted by a strong base to give a carbanion that reacts with aromatic aldehydes to form a compound having a carbon-carbon double bond. Cyclopentanone has been reacted with thiophene-2-carboxaldehyde to yield 2-(2-thienyl)cyclopentanone (Austin *et al.*, 2007; Tsukerman *et al.*, 1964). In the present study, 2-methylcyclopentanone was used in place of the unsubstituted cyclic ketone to yield C_11_H_12_OS (Scheme I); the ketone functionality can be further reacted with, for example, primary amines, to yield other halochromic compounds. The carbon-carbon double-bond i of an *E* configuration. The cyclopentane ring adopts an envelope-shaped conformation whose flap is represented by the methine carbon (Fig. 1). This atom is disordered over two positions in a 90 (1):10 ratio, *i.e.*, it lies above the plane comprising the other non-H atoms in 90% of the molecules, and below the plane in 10% of the molecules.

### Experimental

Thiophene-2-carboxaldehyde (1.10 g, 0.01 mol) in ethanol (20 m) was added to a solution of 2-methylcyclopentanone (0.98 g, 0.01 mol) dissolved in 20% ethanolic potassium hydroxide (20 ml). The mixture was stirred for 6 h. This was then poured into water (200 ml) and set aside for several hours. The precipitated product was collected, washed with water, dried and finallly recrystallized from ethanol to yield faint yellow crystals, 343–343 K.

### Refinement

Carbon-bound H atoms were placed in calculated positions [C—H 0.95–1.00 Å, *U*_iso_(H) = 1.2–1.5*U*_eq_(C)] and were included in the refinement in the riding model approximation.

The methine unit is disordered over two positions with a site occupation factor of 0.899 (8) for the major occupied site. The anisotropic displacement parameters of the primed atom were set to those of the unprimed one, and they were restrained to be nearly isotropic. Pairs of C_methine_—C distances were restrained to within 0.01 Å of each other.

### Crystal data

**Table:**

C_11_H_12_OS	*F*(000) = 408
*M**_r_* = 192.27	*D*_x_ = 1.333 Mg m^−^^3^
Monoclinic, *P*2_1_/*c*	Mo *K*α radiation, λ = 0.71073 Å
Hall symbol: -P 2ybc	Cell parameters from 2335 reflections
*a* = 12.0667 (5) Å	θ = 2.5–29.2°
*b* = 11.0576 (4) Å	µ = 0.29 mm^−^^1^
*c* = 7.3003 (3) Å	*T* = 100 K
β = 100.469 (4)°	Prism, light yellow
*V* = 957.85 (7) Å^3^	0.25 × 0.15 × 0.10 mm
*Z* = 4	

### Data collection

**Table:**

Agilent SuperNova Dual diffractometer with Atlas detector	2131 independent reflections
Radiation source: SuperNova (Mo) X-ray Source	1817 reflections with *I* > 2σ(*I*)
mirror	*R*_int_ = 0.028
Detector resolution: 10.4041 pixels mm^-1^	θ_max_ = 27.5°, θ_min_ = 2.5°
ω scans	*h* = −12→15
Absorption correction: multi-scan (*CrysAlis PRO*; Agilent, 2010)	*k* = −10→14
*T*_min_ = 0.931, *T*_max_ = 0.971	*l* = −9→9
4842 measured reflections	

### Refinement

**Table:**

Refinement on *F*^2^	Primary atom site location: structure-invariant direct methods
Least-squares matrix: full	Secondary atom site location: difference Fourier map
*R*[*F*^2^ > 2σ(*F*^2^)] = 0.041	Hydrogen site location: inferred from neighbouring sites
*wR*(*F*^2^) = 0.104	H-atom parameters constrained
*S* = 0.99	*w* = 1/[σ^2^(*F*_o_^2^) + (0.0433*P*)^2^ + 0.8843*P*] where *P* = (*F*_o_^2^ + 2*F*_c_^2^)/3
2131 reflections	(Δ/σ)_max_ = 0.001
122 parameters	Δρ_max_ = 0.57 e Å^−^^3^
9 restraints	Δρ_min_ = −0.31 e Å^−^^3^

### Fractional atomic coordinates and isotropic or equivalent isotropic displacement parameters (Å^2^)

**Table:**

	*x*	*y*	*z*	*U*_iso_*/*U*_eq_	Occ. (<1)
S1	0.46249 (4)	0.03090 (4)	0.23692 (6)	0.01813 (15)	
O1	0.83453 (11)	0.32223 (13)	0.2085 (2)	0.0294 (4)	
C1	0.32149 (16)	0.06255 (19)	0.1851 (3)	0.0207 (4)	
H1	0.2639	0.0080	0.2046	0.025*	
C2	0.30192 (15)	0.17578 (18)	0.1124 (3)	0.0207 (4)	
H2	0.2287	0.2089	0.0749	0.025*	
C3	0.40176 (14)	0.23853 (17)	0.0985 (2)	0.0172 (4)	
H3	0.4028	0.3183	0.0504	0.021*	
C4	0.49807 (15)	0.17173 (16)	0.1624 (2)	0.0162 (4)	
C5	0.61209 (15)	0.21367 (17)	0.1727 (3)	0.0170 (4)	
H5	0.6195	0.2946	0.1330	0.020*	
C6	0.70943 (15)	0.15444 (17)	0.2306 (3)	0.0181 (4)	
C7	0.81999 (16)	0.21558 (19)	0.2394 (3)	0.0245 (4)	
C8	0.91401 (17)	0.1245 (2)	0.3085 (3)	0.0251 (7)	0.899 (8)
H8	0.9358	0.1353	0.4463	0.030*	0.899 (8)
C8'	0.8943 (7)	0.1127 (8)	0.187 (2)	0.0251 (7)	0.10
H8'	0.8730	0.1011	0.0492	0.030*	0.101 (8)
C9	0.85457 (16)	0.00382 (19)	0.2756 (3)	0.0286 (5)	
H9A	0.8895	−0.0559	0.3697	0.034*	0.899 (8)
H9B	0.8595	−0.0275	0.1503	0.034*	0.899 (8)
H9C	0.9009	−0.0091	0.4006	0.034*	0.101 (8)
H9D	0.8609	−0.0687	0.1988	0.034*	0.101 (8)
C10	0.73056 (16)	0.02545 (17)	0.2918 (3)	0.0197 (4)	
H10A	0.6799	−0.0303	0.2097	0.024*	
H10B	0.7193	0.0143	0.4217	0.024*	
C11	1.01808 (16)	0.1430 (2)	0.2272 (3)	0.0321 (5)	
H11A	1.0501	0.2228	0.2636	0.048*	0.899 (8)
H11B	0.9989	0.1381	0.0911	0.048*	0.899 (8)
H11C	1.0734	0.0802	0.2738	0.048*	0.899 (8)
H11D	1.0276	0.2304	0.2461	0.048*	0.101 (8)
H11E	1.0528	0.1179	0.1219	0.048*	0.101 (8)
H11F	1.0544	0.1004	0.3400	0.048*	0.101 (8)

### Atomic displacement parameters (Å^2^)

**Table:**

	*U*^11^	*U*^22^	*U*^33^	*U*^12^	*U*^13^	*U*^23^
S1	0.0192 (2)	0.0158 (2)	0.0198 (3)	−0.00094 (17)	0.00467 (18)	0.00170 (18)
O1	0.0229 (7)	0.0190 (7)	0.0480 (10)	−0.0013 (6)	0.0112 (7)	0.0039 (7)
C1	0.0185 (9)	0.0236 (10)	0.0199 (9)	−0.0038 (8)	0.0035 (7)	−0.0029 (8)
C2	0.0170 (9)	0.0231 (10)	0.0210 (10)	0.0008 (8)	0.0007 (7)	−0.0021 (8)
C3	0.0192 (9)	0.0165 (9)	0.0151 (9)	−0.0006 (7)	0.0012 (7)	−0.0008 (7)
C4	0.0197 (9)	0.0138 (9)	0.0158 (9)	0.0000 (7)	0.0047 (7)	0.0003 (7)
C5	0.0200 (9)	0.0129 (9)	0.0196 (9)	−0.0007 (7)	0.0081 (7)	0.0008 (7)
C6	0.0191 (9)	0.0159 (9)	0.0205 (9)	−0.0012 (7)	0.0069 (7)	−0.0027 (8)
C7	0.0201 (9)	0.0197 (10)	0.0361 (12)	0.0016 (8)	0.0114 (8)	0.0011 (9)
C8	0.0195 (11)	0.0234 (12)	0.0330 (14)	0.0011 (9)	0.0060 (9)	0.0006 (10)
C8'	0.0195 (11)	0.0234 (12)	0.0330 (14)	0.0011 (9)	0.0060 (9)	0.0006 (10)
C9	0.0195 (9)	0.0220 (10)	0.0413 (13)	0.0022 (8)	−0.0023 (9)	0.0031 (10)
C10	0.0215 (9)	0.0154 (9)	0.0222 (10)	0.0007 (7)	0.0044 (7)	0.0010 (8)
C11	0.0176 (9)	0.0309 (12)	0.0485 (14)	0.0040 (9)	0.0074 (9)	0.0030 (11)

### Geometric parameters (Å, °)

**Table:**

S1—C1	1.7107 (19)	C8—H8	1.0000
S1—C4	1.7293 (18)	C8'—C9	1.487 (9)
O1—C7	1.219 (2)	C8'—C11	1.506 (9)
C1—C2	1.364 (3)	C8'—H8'	1.0000
C1—H1	0.9500	C9—C10	1.541 (3)
C2—C3	1.410 (3)	C9—H9A	0.9900
C2—H2	0.9500	C9—H9B	0.9900
C3—C4	1.384 (3)	C9—H9C	0.9900
C3—H3	0.9500	C9—H9D	0.9900
C4—C5	1.441 (2)	C10—H10A	0.9900
C5—C6	1.344 (3)	C10—H10B	0.9900
C5—H5	0.9500	C11—H11A	0.9800
C6—C7	1.487 (3)	C11—H11B	0.9800
C6—C10	1.503 (3)	C11—H11C	0.9800
C7—C8	1.533 (3)	C11—H11D	0.9800
C7—C8'	1.539 (9)	C11—H11E	0.9800
C8—C11	1.497 (3)	C11—H11F	0.9800
C8—C9	1.513 (3)		
			
C1—S1—C4	92.29 (9)	C7—C8'—H8'	106.8
C2—C1—S1	111.64 (14)	C8'—C9—C10	107.6 (3)
C2—C1—H1	124.2	C8—C9—C10	106.86 (17)
S1—C1—H1	124.2	C8—C9—H9A	110.3
C1—C2—C3	112.96 (17)	C10—C9—H9A	110.3
C1—C2—H2	123.5	C8'—C9—H9B	78.6
C3—C2—H2	123.5	C8—C9—H9B	110.3
C4—C3—C2	112.91 (17)	C10—C9—H9B	110.3
C4—C3—H3	123.5	H9A—C9—H9B	108.6
C2—C3—H3	123.5	C8'—C9—H9C	110.2
C3—C4—C5	125.52 (17)	C10—C9—H9C	110.2
C3—C4—S1	110.19 (13)	C8'—C9—H9D	110.2
C5—C4—S1	124.24 (14)	C10—C9—H9D	110.2
C6—C5—C4	129.13 (17)	H9C—C9—H9D	108.5
C6—C5—H5	115.4	C6—C10—C9	103.84 (15)
C4—C5—H5	115.4	C6—C10—H10A	111.0
C5—C6—C7	121.20 (17)	C9—C10—H10A	111.0
C5—C6—C10	130.35 (17)	C6—C10—H10B	111.0
C7—C6—C10	108.45 (16)	C9—C10—H10B	111.0
O1—C7—C6	126.17 (18)	H10A—C10—H10B	109.0
O1—C7—C8	125.02 (18)	C8—C11—H11A	109.5
C6—C7—C8	108.67 (17)	C8—C11—H11B	109.5
O1—C7—C8'	124.0 (4)	H11A—C11—H11B	109.5
C6—C7—C8'	102.2 (4)	C8—C11—H11C	109.5
C11—C8—C9	117.7 (2)	C8'—C11—H11C	119.9
C11—C8—C7	113.82 (19)	H11A—C11—H11C	109.5
C9—C8—C7	103.08 (16)	H11B—C11—H11C	109.5
C11—C8—H8	107.2	C8'—C11—H11D	109.5
C9—C8—H8	107.2	H11B—C11—H11D	101.3
C7—C8—H8	107.2	C8'—C11—H11E	109.5
C9—C8'—C11	118.8 (8)	H11A—C11—H11E	105.3
C9—C8'—C7	104.0 (6)	H11D—C11—H11E	109.5
C11—C8'—C7	112.9 (7)	C8'—C11—H11F	109.5
C9—C8'—H8'	106.8	H11D—C11—H11F	109.5
C11—C8'—H8'	106.8	H11E—C11—H11F	109.5
			
C4—S1—C1—C2	0.45 (16)	O1—C7—C8'—C9	172.1 (4)
S1—C1—C2—C3	−0.2 (2)	C6—C7—C8'—C9	−36.9 (9)
C1—C2—C3—C4	−0.2 (2)	C8—C7—C8'—C9	68.5 (8)
C2—C3—C4—C5	−177.31 (17)	O1—C7—C8'—C11	42.1 (12)
C2—C3—C4—S1	0.5 (2)	C6—C7—C8'—C11	−167.0 (7)
C1—S1—C4—C3	−0.56 (15)	C8—C7—C8'—C11	−61.6 (8)
C1—S1—C4—C5	177.32 (16)	C11—C8'—C9—C8	59.1 (9)
C3—C4—C5—C6	−179.18 (19)	C7—C8'—C9—C8	−67.4 (8)
S1—C4—C5—C6	3.3 (3)	C11—C8'—C9—C10	153.4 (7)
C4—C5—C6—C7	−177.08 (18)	C7—C8'—C9—C10	27.0 (9)
C4—C5—C6—C10	3.9 (3)	C11—C8—C9—C8'	−58.8 (6)
C5—C6—C7—O1	4.8 (3)	C7—C8—C9—C8'	67.4 (6)
C10—C6—C7—O1	−176.0 (2)	C11—C8—C9—C10	−155.4 (2)
C5—C6—C7—C8	−179.48 (18)	C7—C8—C9—C10	−29.2 (2)
C10—C6—C7—C8	−0.3 (2)	C5—C6—C10—C9	161.5 (2)
C5—C6—C7—C8'	−145.3 (6)	C7—C6—C10—C9	−17.6 (2)
C10—C6—C7—C8'	33.9 (6)	C8'—C9—C10—C6	−6.5 (7)
O1—C7—C8—C11	−37.2 (3)	C8—C9—C10—C6	29.4 (2)
C6—C7—C8—C11	146.97 (19)	C9—C8—C11—C8'	57.6 (6)
C8'—C7—C8—C11	63.0 (6)	C7—C8—C11—C8'	−63.1 (6)
O1—C7—C8—C9	−165.9 (2)	C9—C8'—C11—C8	−60.2 (9)
C6—C7—C8—C9	18.3 (2)	C7—C8'—C11—C8	61.9 (8)
C8'—C7—C8—C9	−65.7 (5)		
